# A Russian Doll of Resistance: Nested Gains and Losses of Venom Immunity in Varanid Lizards

**DOI:** 10.3390/ijms25052628

**Published:** 2024-02-23

**Authors:** Uthpala Chandrasekara, Marco Mancuso, Lorenzo Seneci, Lachlan Bourke, Dane F. Trembath, Joanna Sumner, Christina N. Zdenek, Bryan G. Fry

**Affiliations:** 1 Adaptive Biotoxicology Lab, School of the Environment, University of Queensland, St Lucia, QLD 4072, Australia; u.chandrasekara@uq.net.au (U.C.); 19marcomancuso19@gmail.com (M.M.); l.seneci@uq.net.au (L.S.); l.bourke@uq.net.au (L.B.); 2 Herpetology Department, Australian Museum Research Institute, Australian Museum, Sydney, NSW 2010, Australia; dane.trembath@australian.museum; 3 Museums Victoria Research Institute, Melbourne, VIC 3001, Australia; jsumner@museum.vic.gov.au; 4 School of the Environment, University of Queensland, St Lucia, QLD 4072, Australia; c.zdenek@uq.edu.au

**Keywords:** venom, resistance, evolution, varanid, neurotoxin, nicotinic acetylcholine receptor

## Abstract

The interplay between predator and prey has catalyzed the evolution of venom systems, with predators honing their venoms in response to the evolving resistance of prey. A previous study showed that the African varanid species *Varanus exanthematicus* has heightened resistance to snake venoms compared to the Australian species *V. giganteus*, *V. komodoensis*, and *V. mertensi*, likely due to increased predation by sympatric venomous snakes on *V. exanthematicus*. To understand venom resistance among varanid lizards, we analyzed the receptor site targeted by venoms in 27 varanid lizards, including 25 Australian varanids. The results indicate an active evolutionary arms race between Australian varanid lizards and sympatric neurotoxic elapid snakes. Large species preying on venomous snakes exhibit inherited neurotoxin resistance, a trait potentially linked to their predatory habits. Consistent with the ‘use it or lose it’ aspect of venom resistance, this trait was secondarily reduced in two lineages that had convergently evolved gigantism (*V. giganteus* and the *V. komodoensis*/*V. varius* clade), suggestive of increased predatory success accompanying extreme size and also increased mechanical protection against envenomation due to larger scale osteoderms. Resistance was completely lost in the mangrove monitor *V. indicus*, consistent with venomous snakes not being common in their arboreal and aquatic niche. Conversely, dwarf varanids demonstrate a secondary loss at the base of the clade, with resistance subsequently re-evolving in the burrowing *V. acanthurus*/*V. storri* clade, suggesting an ongoing battle with neurotoxic predators. Intriguingly, within the *V. acanthurus*/*V. storri* clade, resistance was lost again in *V. kingorum*, which is morphologically and ecologically distinct from other members of this clade. Resistance was also re-evolved in *V. glebopalma* which is terrestrial in contrast to the arboreal/cliff dwelling niches occupied by the other members of its clade (*V. glebopalma*, *V. mitchelli*, *V. scalaris*, *V. tristis*). This ‘Russian doll’ pattern of venom resistance underscores the dynamic interaction between dwarf varanids and Australian neurotoxic elapid snakes. Our research, which included testing *Acanthophis* (death adder) venoms against varanid receptors as models for alpha-neurotoxic interactions, uncovered a fascinating instance of the Red Queen Hypothesis: some death adders have developed more potent toxins specifically targeting resistant varanids, a clear sign of the relentless predator–prey arms race. These results offer new insight into the complex dynamics of venom resistance and highlight the intricate ecological interactions that shape the natural world.

## 1. Introduction

### 1.1. Venom Evolution and Predator–Prey Dynamics

Venom, as an ecological trait, evolves through the selection pressures of predator–prey interactions, aiding in the diversification of venomous systems [1]. This evolutionary arms race enhances toxin affinity for pathophysiological targets, increasing the prey’s vulnerability and facilitating their capture [2]. However, in response to the threat posed by venom, prey species have evolved resistance mechanisms, as have the predators of venomous species, diminishing their sensitivity to the toxins and lessening the detrimental effects of envenomation [3]. This may be accomplished through a myriad of methods including modifying the target to reduce toxin binding affinity [4,5,6,7,8,9]. This co-evolutionary process is driven by the reciprocal selection pressures that venom constituents exert on prey species, leading to ongoing adaptive changes consistent with the Red Queen Hypothesis [10,11,12,13]. Such dynamic evolutionary battles between venomous predators and their prey result in a continuous flux of phenotypic and genotypic diversity within these species [14,15].

### 1.2. Mechanisms of Resistance to Snake Venom α-Neurotoxins

Various animals cohabitating with neurotoxically venomous elapid snakes, whether as prey or predators, have developed significant resistance to α-neurotoxic three-finger peptides [4,5,16,17,18,19,20,21,22,23,24]. These toxins predominantly target the postsynaptic muscle-type α-1 nicotinic acetylcholine receptors (nAChR), producing flaccid paralysis by disrupting the crucial nerve–muscle signal transmission [1]. Consequently, a strong selection pressure exists for evolutionary adaptations conferring resistance to these neurotoxins within the nAChRs themselves. As such, the primary resistance mechanism involves alterations to the ligand-binding domain of the postsynaptic muscle-type α-1 nAChR, whereby specific amino acid changes at the orthosteric site reduce receptor sensitivity to 3FTxs while maintaining its affinity for the natural neurotransmitter, acetylcholine [3]. Intriguingly, there is a ‘use it or lose it’ pattern demonstrated by prey species which have radiated out of the range of venomous predators, with a subsequent secondary loss of resistance [25]. This suggests a fitness disadvantage in that the acetylcholine binding is less efficient in resistant species, with this therefore subjected to purifying selection pressure in the absence of a correspondingly larger advantage gained by resistance to sympatric venomous species [25]. 

Two primary forms of α-neurotoxicity resistance have been described: steric hindrance, where toxin binding is physically blocked by receptor modifications; and electrochemical repulsion, where similar charges on the toxin and receptor lead to mutual repulsion. These evolutionary modifications are critical for the continued survival of species in ecosystems where neurotoxic snakes are prevalent predators.

Steric hindrance was first described in the Egyptian Mongoose (*Herpestes ichneumon*), a predator of cobras [4], and subsequently shown to be a basal trait in the snake-eating family Herpestidae [25,26]. This form of resistance has been shown to have convergently evolved widely in both prey and predators of neurotoxic venomous snakes, including within the snakes themselves as a resistance against their own venoms [19,25,27]. This form of resistance evolves by mutations, typically at orthosteric positions 187 or 189, that confer a structural alteration at the orthosteric site. Here, the original amino acid is substituted with an asparagine (N) residue that undergoes post-translational modification, acquiring a bulky glycosylation side chain. This large glycan moiety on the asparagine (N) likely acts as a physical barrier, obstructing the attachment of α-neurotoxins to the receptor site and thus conferring resistance to the venom. A second form of steric hindrance resistance to neurotoxic venom has also been observed across various animal groups, involving the substitution of the orthosteric site’s proline (P) at positions 194 and 197 [16]. Proline is known to induce a distinct bend in the peptide chain, which is critical for maintaining the protein’s normal secondary structure. Consequently, when mutations lead to the replacement of proline with alternative amino acids, the conformation of the nAChR’s orthosteric site is structurally altered, impeding the ability of α-neurotoxins to bind effectively to the receptor.

Similar to the mechanism of steric hindrance, the electrostatic charge-repulsion form of resistance is also documented in both the natural prey and the predatory species of venomous snakes [22,26,28]. Instances of this resistance have been identified as convergently evolving in diverse creatures, including snake predators such as the honey badger (*Mellivora capensis*), various hedgehog species (*Erinaceus concolor* and *Erinaceus europaeus*), pigs (*Sus scrofa*), and slow moving prey of sympatric snakes such as the Burmese python, the mole snake, and caecilians [22,26,27,28]. In the above-mentioned predatory mammals, an ancestral amino acid, tryptophan (W), at position 187 of the orthosteric site, is replaced by a positively charged arginine (R), with this single mutation responsible for the iconic resistance of honey badgers against cobra venom [22,26,28]. In the prey species, negatively charged amino acids aspartic acid (D) or glutamic acid (E) at positions 191 or 195 (or both) have been discovered to be replaced by the positively charged lysine (K), conferring strong resistance to elapid venoms [22,27]. Even the loss of the negatively charged amino acids without replacement by positively charged amino acids decreases the binding of affinity of the neurotoxic venom peptides [24], consistent with the strong positive charge on the surface of the toxins being attracted to the negative charges in the orthosteric site as the initial guide towards binding [2]. Therefore, while introducing positive charges at any point on the receptor site leads to electrostatic repulsion, the loss of the negatively charged amino acids is enough to confer decreased affinity of the positively charged neurotoxins for the receptor. However, this repulsion is more pronounced when negative charges at the receptor sites 191 or 195 (or both) are replaced with positive ones, sharply enhancing the organism’s resistance to the venom.

### 1.3. Varanid Lizard Diversity and Dietary Patterns

*Varanus*, the genus encompassing the varanid lizards, boasts a broad distribution across Asia, Africa, and Australia. This genus is remarkable for its significant morphological similarities across species while exhibiting vast differences in size and mass [29]. Australia has the greatest range of varanid sizes, spanning from the diminutive *V. sparnus*, measuring less than 20 cm in length and weighing less than 10 g, to the colossal *V. giganteus*, which can reach lengths of 2 m and weigh around 17 kg [30]. The Australian varanid radiation is considered a striking example of adaptive evolution, with two distinct evolutionary lineages leading to a wide array of sizes: the subgenus *Varanus* growing into large or gigantic sizes, as is the basal condition globally for the genus, and the Australian endemic subgenus *Odatria* evolving into dwarf forms. Varanids have diversified into a range of ecological niches, including aquatic, terrestrial, arboreal, semi-arboreal, and fossorial lifestyles [31]. These active predators generally have a generalized diet, consuming mainly invertebrates and occasionally vertebrates like reptiles (including venomous snakes), rodents, small mammals, and frogs [31,32].

**Figure 1:**
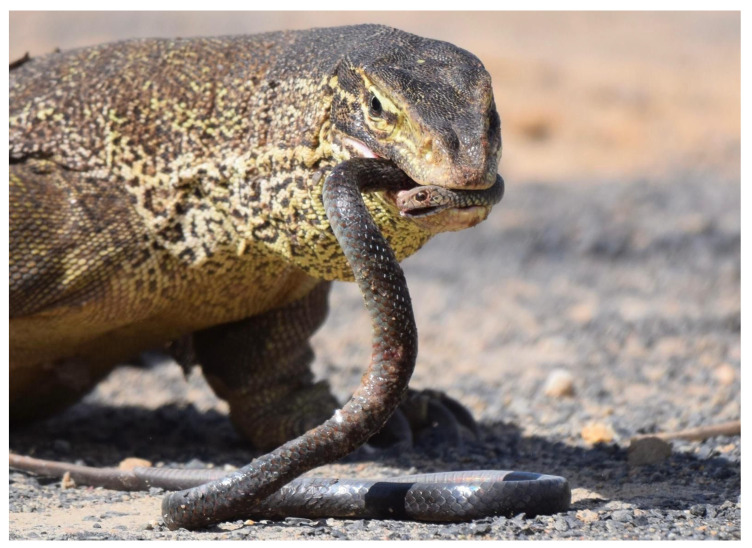
*Varanus panoptes* (Yellow-spotted Monitor) preying upon the elapid snake species *Demansia vestigiata* (Black Whipsnake). Photo by James Dobson.

Notably, in Australia, there is an extraordinary diversity of reptiles, including a high number of venomous snakes predominantly from the Elapidae family [33]. Given the abundance and diversity of these venomous snakes, we hypothesize that varanid lizards subject to predation, or that prey on elapids (Figure 1), may have evolved mechanisms for reduced susceptibility to their neurotoxins. Prior research revealed that the African *V. exanthematicus*, a slow-moving species vulnerable to predation by the abundant sympatric cobras, exhibits resistance through specific amino acid motifs in the nAChR orthosteric site [24]. This supports the possibility of similar evolutionary adaptations in Australian varanid species. Conversely, it is hypothesized that Australian varanids less threatened by neurotoxic elapids, due to morphological, behavioral, or geographical advantages, will not possess such resistance motifs in their nAChRs due to the fitness disadvantage imparted by modified receptors, in the absence of a corresponding fitness advantaged gained by reduced levels of envenomations by neurotoxic predators or prey.

### 1.4. Study Aims and Approach

This study aimed to explore the adaptive strategies of varanid lizards, particularly those susceptible to predation by, or who are predators of, neurotoxic elapids. To achieve this, we sequenced the muscular nAChR ligand-binding regions of various Australian varanids for comparative analysis. The sequences were scrutinized for steric and electrostatic resistance motifs using an Octet-based biolayer interferometry assay. This assay assessed venom-binding affinities across eight potently α-neurotoxic *Acanthophis* species from different regions, applying native and engineered varanid orthosteric sites. *Acanthophis* venoms were chosen as model venoms as they are overwhelmingly alpha-neurotoxic, in contrast to other Australian elapids which may be mixtures of alpha-neurotoxic, presynaptic neurotoxic, and non-neurotoxic pathophysiological actions including coagulotoxicity and myotoxicity [34]. Additionally, we employed maximum-likelihood selection tests to pinpoint positively selected sites influenced by the evolutionary pressure of snake venom α-neurotoxins. Ancestral character states were reconstructed to determine if α-neurotoxin resistance is an inherited trait across the Varanidae family or a product of convergent evolution specific to varanids frequently exposed to neurotoxic elapids. This investigation is pioneering in studying α-neurotoxin resistance within Australian varanids, thus bridging a significant gap in the current understanding of their defense mechanisms against native elapids. The outcomes will delineate the structural motifs in the α-1 nAChR orthosteric site that impart resistance to α-neurotoxins, shedding light on the intricate dynamics of predator–prey interactions.

## 2. Results

### 2.1. Orthosteric Site Characterization

The defining feature of the α1-nAChR orthosteric site is a segment of 14 amino acids, spanning regions 187–200. In an expansion of research on Australian varanids, which previously included only a limited number of species [24], the α1-nAChR orthosteric site was sequenced from 23 species, representing a wide taxonomic and ecological spectrum. We complemented this data with orthosteric sequences from two Australian species, along with one African and one Asian species, sourced from the NCBI database, bringing the total to 27 varanid species examined for ligand-binding domain sequences of α1-nAChR. The investigation’s goal was to ascertain the relative specific amino acid motifs within the orthosteric sites that are known to confer resistance to α-neurotoxins. 

**Figure 2:**
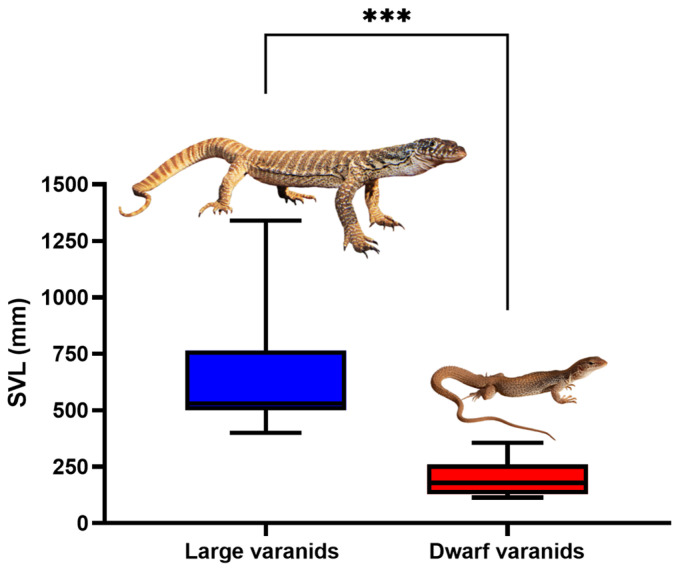
Box-and-whisker plot showing the variation in size between large (SVL > 500 mm, represented) and small (SVL < 500 mm) varanid species. Photo credits: Daniel Bromely (*V. spenceri*); Christina N Zdenek (*V. storri*). *** indicates statistically significant differences.

There were differential patterns in relative conservation or diversification, both at amino acid position and varanid lizard clade. Notably, all varanid orthosteric sites analyzed shared a conserved disulfide bridge formed by two cysteine residues at positions 192–193, a critical element in shaping the ligand-binding pocket’s structure [21,35,36]. Additionally, highly conserved tyrosine residues at positions 190 and 198 [37] were present in all varanid species assessed, irrespective of their geographic evolution, hinting at its potential critical role in anchoring the endogenous neurotransmitter acetylcholine within the receptor’s orthosteric site. In contrast to the highly conserved amino acids at positions 190, 192, 193, and 198, the sequence analysis conducted in this research has revealed significant variations at the orthosteric sites, specifically at positions 187, 189, 191, 195, and 196, among the varanid lizards studied. These variations were notably different between the two distinct morphological groups (Figure 2): the clade of large Australian varanids, which are morphologically similar to Asian and African varanids and as such represent the basal morphotype; and the morphologically derived dwarf varanid species (*Odatria* subclade). This pattern suggests a divergence in the amino acid composition at the orthosteric sites, which may correlate with the physical size and ecological adaptations of these two varanid clades.

**Figure 3:**
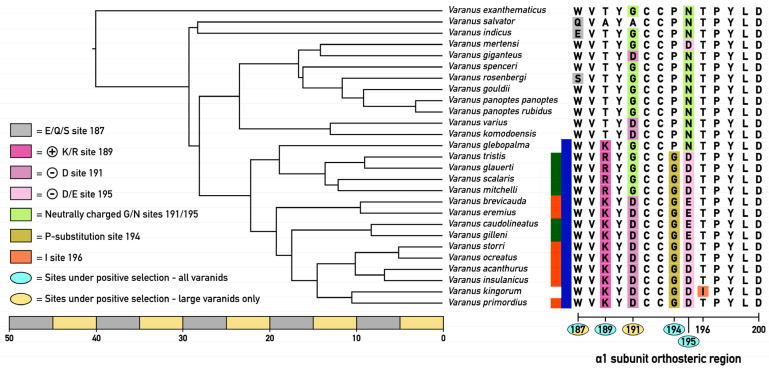
The ancestral state reconstruction of varanid lizards showing the amino acid motifs of the 187–200 region in the α1-nAChR ligand-binding domain. The phylogenetic tree was constructed from Timetree.org, modified in Figtree, and imported into Inkscape where additional manual editing was performed based up the most recent phylogenomic taxonomy [29,40]. Sites showing significant positive selection are highlighted in different colors for the relevant species. Deviations from the ancestral amino acids at sites 187 are grey. The evolution of positively charged amino acids in dwarf varanids has been colored in dark pink. The green-colored amino acids at 191 and 195 orthosteric sites implies the resistance granted by neutrally charged amino acids at the key orthosteric sites. Negatively charged amino acids (glutamic acid, aspartic acid) at the same sites (191/195) are represented in shades of light pink. Ochre-colored boxes at site 194 represent substitutions of prolines. Isoleucine substituting the evolutionary conserved threonine at position 196 in *V. kingorum* is displayed in orange. Blue vertical bar indicates the dwarf varanid clade. Green vertical bars indicate the arboreal dwarf varanids. Red vertical bars indicate burrowing dwarf varanids.

The large varanid lizards, which represent the basal morphology, are distributed across Africa, Asia, and Australia [30,38], have adult sizes typically exceeding one meter in length (Figure 2). Species included in this study were *V. exanthematicus*, *V. giganteus*, *V. gouldii*, *V. indicus*, *V. komodoensis*, *V. mertensi*, *V. panoptes*, *V. panoptes rubidus*, *V. spenceri, V. rosenbergii*, *V. salvator*, and *V. varius*. Recognized as upper trophic-level predators within their ecosystems, the large varanids often engage in predatory behaviors towards other sympatric species, including neurotoxic elapid snakes [30,31,39]. This predatory dynamic posits a hypothesis that these varanids may have developed adaptive traits for α-neurotoxin resistance, specifically within the α1-nAChR orthosteric sites of their receptors. Such adaptations would be evolutionary responses to mitigate the risks associated with neurotoxic envenomation during predatory encounters, thus potentially increasing their survival against α-neurotoxic snake venom. In the larger Australian varanids, the orthosteric sites revealed a consistent pattern of amino acid sequences that align closely with those found in their African and Asian counterpart (Figure 3). As such, paralleling the large size being the basal trait, the larger varanids’ orthosteric sites are the ancestral type, with the basal condition including resistance motifs that have been previously experimentally validated for varanid species [24].

On the other hand, the orthosteric sequences of the smaller Australian varanids have diverged significantly from this ancestral model, displaying a variety of mutations (Figure 3). These mutations have led to the emergence of amino acid residues at crucial biochemical sites, which are known to be pivotal for the binding of α-neurotoxins. Paralleling this, the clade of dwarf varanid lizards represents the derived morphological condition, typically not exceeding one meter in length at maturity. This clade, endemic to Australia, encompasses a diverse group, including the study species: *V. acanthurus*, *V. brevicauda*, *V. caudolineatus*, *V. eremius*, *V. gilleni*, *V. glauerti*, *V. glebopalma*, *V. insulanicus*, *V. kingorum*, *V. mitchelli*, *V. primordius*, *V. scalaris*, *V. ocreatus*, *V. storri*, and *V. tristis*. Given their diminutive size, dwarf varanids are potentially more vulnerable to predation by sympatric neurotoxin-producing elapids such as *Acanthophis* species (death adders). We hypothesize that such size-related predation risks may exert a selection pressure for these dwarf varanids to develop defensive adaptations within the α1-nAChR orthosteric sites to mitigate the impact of α-neurotoxins present in elapid venom. This evolutionary hypothesis suggests a correlation between predator–prey dynamics and molecular adaptations in neurotoxin resistance.

### 2.2. Functional Validation of Resistance Motifs

To test the hypotheses regarding selection pressure on varanids that are predators, and varanids that are prey, and to investigate the role of novel amino acid substitutions in varanid orthosteric sites for α-neurotoxin resistance, we measured the binding of α-neurotoxins to the α1-nAChR orthosteric sites using our validated biolayer interferometry technique [24,41,42]. This innovative peptide mimotope-based approach significantly enhances our understanding of receptor-toxin affinities on a taxon-specific level, offering a novel way to explore the interactions of these potent toxins with their biological targets, particularly nAChRs.

**Figure 4:**
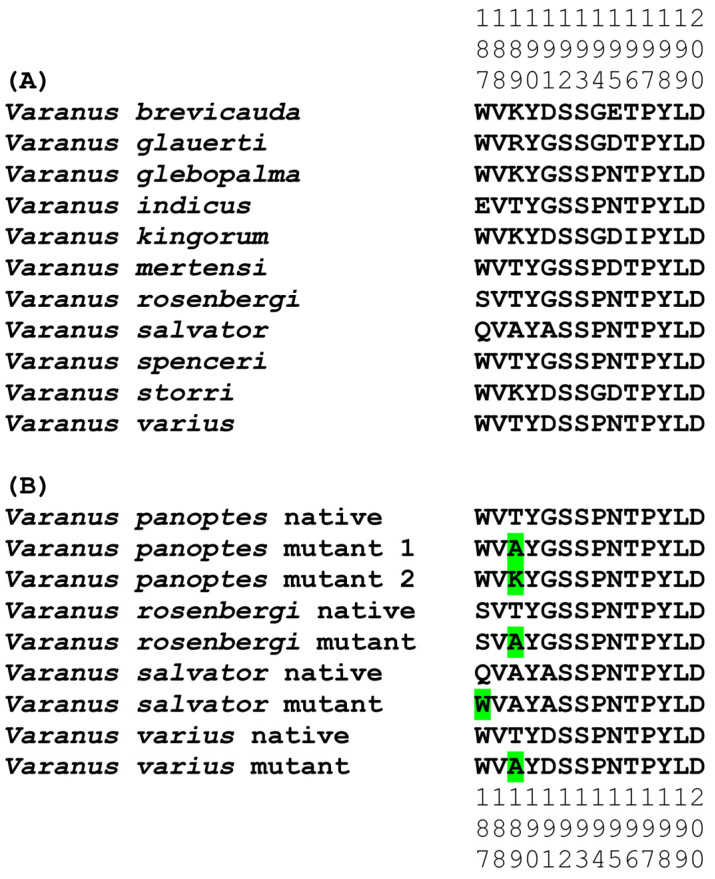
(**A**) Amino acid sequences at positions 187–200 of native varanid orthosteric sites of nAChR. (**B**) Amino acid swap mutants of the varanid orthosteric sites. The substituted amino acids are highlighted in green. Note: As per Section 4.5, the cysteine-doublet is replaced during peptide synthesis by a serine doublet to prevent uncontrolled postsynthetic thiol oxidation [43]. As per Figure 3, some mimotopes are identical across multiple species: ***V. brevicauda*** = *V. caudolineatus*, *V. eremius*, *V. gilleni*; ***V. glauerti*** = *V. mitchelli*, *V. scalaris*, *V. tristis*; ***V. spenceri*** = *V. exanthematicus*, *V. gouldii*, *V. panoptes panoptes*, *V. panoptes rubidus*; ***V. storri*** = *V. acanthurus*, *V. insulanicus*, *V. primordius*, *V. ocreatus*; ***V. varius*** = *V. giganteus*, *V. komodoensis*.

In the initial phase, we synthesized a collection of native peptide mimotopes, each comprising 14 amino acids, reflecting the orthosteric sites spanning amino acid positions 187–200 on the α1 subunit of the nAChR for each varanid species (Figure 4A). As some orthosteric sites were identical between closely related species (Figure 3), the final set was narrowed down to 11 distinct varanid sequences for the creation of native mimotope peptides. Following this, we constructed a series of mutant mimotopes by altering specific amino acids to ascertain those responsible for conferring resistance to neurotoxic venom (Figure 4B).

### 2.3. Sequence Analysis and Hypothesis Testing Using Biolayer Interferometry

#### 2.3.1. Orthosteric Site Position 187

**Figure 5:**
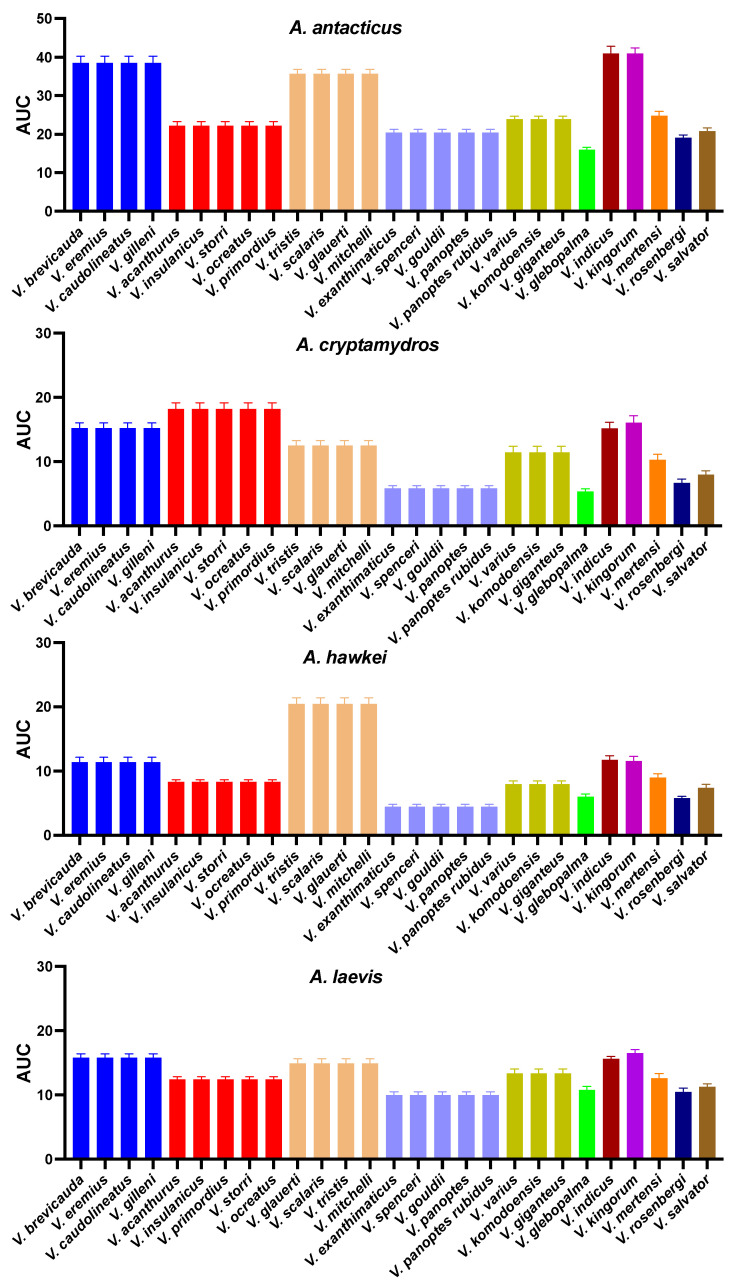
Bar graphs of the area under curve showing a comparison of the venom binding affinities of the varanid native peptide mimotopes against eight *Acanthophis* species’ venom. Amino acid sequences at positions 187–200 of native varanid lizards’ orthosteric sites. Species from the same clade are shown with the same color. Groupings are by mimotope. As per Figure 3, some mimotopes are identical across multiple species: ***V. brevicauda*** = *V. caudolineatus*, *V. eremius*, *V. gilleni*; ***V. glauerti*** = *V. mitchelli*, *V. scalaris*, *V. tristis*; ***V. spenceri*** = *V. exanthematicus*, *V. gouldii*, *V. panoptes panoptes*, *V. panoptes rubidus*; ***V. storri*** = *V. acanthurus*, *V. insulanicus*, *V. primordius*, *V. ocreatus*; ***V. varius*** = *V. giganteus*, *V. komodoensis*.

**Figure 6:**
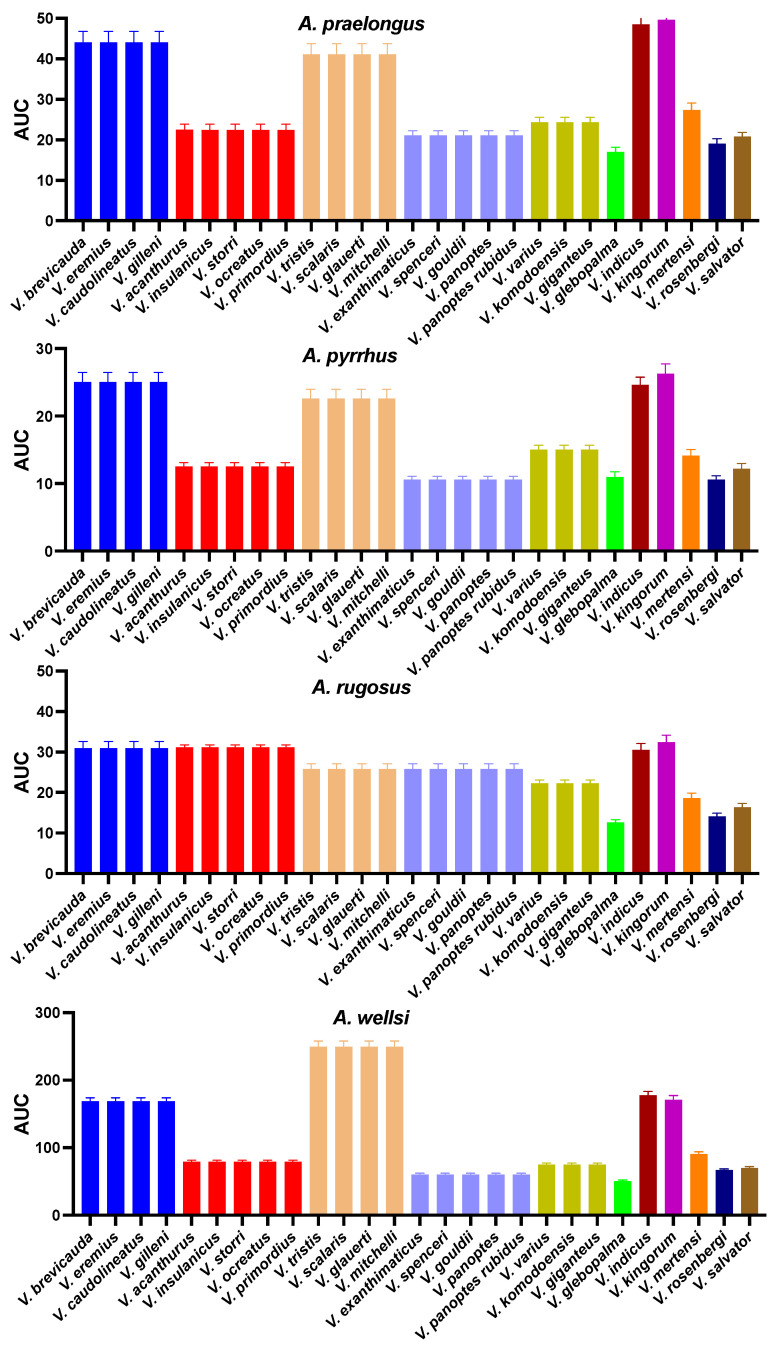
Bar graphs of the area under curve showing a comparison of the venom binding affinities of the varanid native peptide mimotopes against eight *Acanthophis* species’ venom. Amino acid sequences at positions 187–200 of native varanid lizards’ orthosteric sites. Species from the same clade are shown with the same color. Groupings are by mimotope. As per Figure 3, some mimotopes are identical across multiple species: ***V. brevicauda*** = *V. caudolineatus*, *V. eremius*, *V. gilleni*; ***V. glauerti*** = *V. mitchelli*, *V. scalaris*, *V. tristis*; ***V. spenceri*** = *V. exanthematicus*, *V. gouldii*, *V. panoptes panoptes*, *V. panoptes rubidus*; ***V. storri*** = *V. acanthurus*, *V. insulanicus*, *V. primordius*, *V. ocreatus*; ***V. varius*** = *V. giganteus*, *V. komodoensis*.

**Figure 7:**
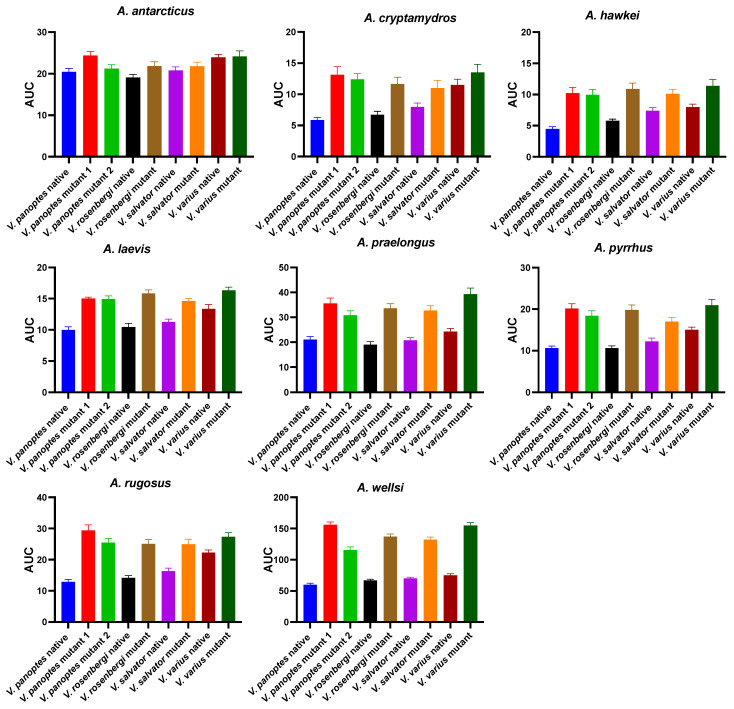
Bar graphs of the area under curve showing a comparison of the venom binding affinities of the native and mutant peptide mimotopes against eight *Acanthophis* species’ venoms. As per Figure 3, the *V. varius* mimotope is shared with *V. komodoensis*, and identically convergently evolved in *V. giganteus*.

The majority of tested varanids retained the ancestral tryptophan (W) (Figure 3). None of the species studied had the arginine substitution at this position previously identified as conferring the high level of resistance evident in the honey badger [22]. However, we identified three novel mutations at orthosteric position 187, independently emerging in *V. indicus*, *V. rosenbergi*, and *V. salvator*, where tryptophan (W) at position 187 is replaced with glutamic acid (E), serine (S), and glutamine (Q), respectively (Figure 3). Our observations revealed that the native *V. indicus* orthosteric site was strongly bound by all *Acanthophis* venoms tested (Figure 5 and Figure 6). In contrast, the native *V. spenceri* orthosteric site, which is identical to the native *V. indicus* mimotope except for the presence of ancestral tryptophan (W) at 187 in *V. spenceri*, whereas *V. indicus* has glutamic acid (E) at 187, demonstrated significantly lower binding by all *Acanthophis* venoms. This supports the hypothesis that the heightened α-neurotoxin binding observed in *V. indicus* is solely attributed to the presence of negatively charged glutamic acid (E) at position 187. This underscores the importance of orthosteric position 187 as a crucial site in α-neurotoxin binding, with the negatively charged amino acid at position 187 being highly susceptible to binding with positively charged α-neurotoxins. However, as *V. indicus* lives in mangrove forests, it is not likely to frequently encounter neurotoxic elapids as potential prey items, and thus the lessened binding by neurotoxic venoms is consistent with the lack of neurotoxic prey featuring in their diet. In contrast, the native *V. salvator* orthosteric site was not strongly bounded by neurotoxic venoms (Figure 5 and Figure 6). However, a *V. salvator* mutant, which was constructed by swapping the amino acid glutamine (Q) of orthosteric position 187 of native *V. salvator* mimotope with ancestral tryptophan (W), had a notable increase of binding was observed with mutant mimotope towards all of the *Acanthophis* venoms tested (Figure 7), indicating the fact that glutamine (Q) at orthosteric position 187 of *V. salvator* might be substantially contributing to the reduced α neurotoxin susceptibility. While like *V. indicus*, *V. salvator* is semi-aquatic, it is otherwise terrestrial and would therefore be likely to have frequent encounters with neurotoxic prey. Therefore, the mutation at position 187 in *V. indicus* is neutral, and does not affect fitness, while the mutation at the same position in *V. salvator* is indicative of a fitness advantage by conferring resistance against neurotoxic prey. This conclusion was supported by the statistically significant *p*-values for all venoms tested (Table 1). This amino acid position was also neutral for *V. rosenbergi* as the venom binding was the same for this species as it was for *V. gouldii*, which differed only by having the ancestral W in this position. Therefore, while the mutation in *V. salvator* conferred resistance, it was not involved in resistance in either *V. indicus* or *V. rosenbergi.*

#### 2.3.2. Orthosteric Site Position 189

The receptor–ligand binding analysis of a previous work revealed mutations resulting in positively charged amino acids at orthosteric position 189 contributes to α-neurotoxin resistance in Burmese python but to a lesser degree than such charge-reversal mutations at positions 191 and 195 [22]. Position 189 of the orthosteric sequence from all the larger Australian monitor lizards retains the ancestral polar, uncharged amino acid threonine (T) (Figure 3) [25]. The ancestral threonine (T) is not targeted neurotoxins, as evidenced by mutations of the *V. panoptes* and *V. rosenbergi* sequences whereby T was swapped for either the nonpolar, aliphatic α-amino acid alanine or the positively charged lysine, both of which produced an increase in binding (Figure 7). As such, the consistent mutations in the small Australian varanids to positively charged amino acids (lysine or arginine) (Figure 3), is not indicative of evolution of venom resistance and is instead enigmatic in light of the role in acetylcholine binding [16].

#### 2.3.3. Orthosteric Site Position 191

This position is naturally highly variable [25], but the majority of species have the negatively charged aspartic acid (D) at this position, which has been shown to be highly sensitive to binding by snake venom neurotoxins [22,24]. With the exception of the massively built species *V. giganteus*, *V. komodoensis*, and *V. varius*, all the large varanids had the neutral small amino acid glycine at this position, consistent with their lessened sensitivity to neurotoxins venoms (Figure 5 and Figure 6). The change in venom affinity was shown to be statistically significant (Table 1). As *V. giganteus* is not phylogenetically sister to the *V. komodoensis*/*V. varius* clade (Figure 3), it is unclear if the mutation conferring the loss of aspartic acid (D) at position 191 occurred at the base of the varanid radiation, and was regained on three occasions (once in *V. giganteus*, again in the last common ancestor of *V. komodoensis*, and *V. varius*, and again in the last common ancestor of the dwarf varanids) or aspartic acid (D) was present at position 191 in the last common ancestor and was subsequently lost on at least five independent occasions. Based on the phylogenetic positioning (Figure 3) the most parsimonious explanation is that a glycine was present at position 191 in the varanid last common ancestor, and that on three separate occasions a mutation occurred at position 191 to confer aspartic acid (D), and that in *V. salvator* a separate mutation confers the alanine at this position. 

#### 2.3.4. Orthosteric Site Position 194

While all of the dwarf Australian varanid clade possess the modification to the proline (P) subsite at orthosteric position 194 that has been hypothesized to be associated with neurotoxin resistance [16,22] (Figure 3), this was no pattern of resistance evident for these varanids. Therefore, if mutations at this site are indeed involved in resistance, it is a complex scenario involving interactions with other amino acids. 

#### 2.3.5. Orthosteric Site Position 195

Consistent with previous results [22,24], venom resistance was noted for species that had mutations at position 195, particularly for large varanid species with mutations at both 191 and 195, However, none of the species had charge reversal mutations at this position or 191. Species with only mutations at 195 but not 191 (*V. giganteus*, *V. komodoensis*, and *V. varius*) displayed lower levels of resistance than those with mutations at both positions. This is consistent with the higher α-neurotoxin binding seen in smaller Australian varanids which had negatively charged amino acids at both positions. The change in venom affinity was shown to be statistically significant (Table 1).

There is, however, a conspicuous variation in neurotoxin binding within the dwarf varanids. Whereby those with aspartic acid (D) at 191 and glutamic acid (E) at 195 are bound more strongly by neurotoxins than those with aspartic acid (D) at both 191 and 195. The phylogenetic positioning (Figure 3) suggests that the last common ancestor of the dwarf varanids had a glycine (G) at 191 and aspartic acid (D) at 195. As such, the aspartic acid (D) at position 191 evolved in the last common ancestor of the clade spanning *V. brevicauda*/*V. primordius* (Figure 3). Further, this interpretation suggests that the glutamic acid (E) at position 195 evolved on two convergent occasions: once in the last common ancestor of *V. brevicauda* and *V. eremius*, and again in the last common ancestor of *V. caudolineatus* and *V. gilleni* (Figure 3). While the evolutionary significance of this is unclear, it is conspicuous that the clade spanning *V. storri*/*V. primordius* (Figure 3) are notably less sensitive to *Acanthophis* venoms than the other dwarf varanids (Figure 5 and Figure 6) with the exception of *V. kingorum* (see position 196 below). The change in venom affinity was shown to be statistically significant (Table 1). 

The *V. storri*/*V. primordius* clade (Figure 3) are terrestrial burrowers in hard pack, rocky soil, in contrast to the *V. brevicauda*/*V. eremius* clade (Figure 3) which are burrowers in soft sandy soil. The *V. glebopalma*/*V. mitchelli* clade are arboreal species, with the exception of *V. mitchelli* which is arboreal/semi-aquatic. The habitat favored by the *V. storri*/*V. primordius* clade is conspicuously denser with larger neurotoxic venomous snakes than that of the *V. brevicauda*/*V. eremius* clade (which has smaller neurotoxic species not able to predate upon these varanids). In contrast, Australia lacks arboreal neurotoxic elapid snakes, and as such the *V. glebopalma*/*V. mitchelli* clade would not be prey to neurotoxic snakes. 

#### 2.3.6. Orthosteric Site Position 196

A novel mutation was identified in dwarf varanid *V. kingorum*, where the conserved amino acid residue threonine (T) at orthosteric position 196 in the *V. storri*/*V. primordius* clade (Figure 3) is replaced with isoleucine (I). This single mutation results in *V. kingorum* being less resistant to neurotoxins (Figure 5 and Figure 6). The change in venom affinity was shown to be statistically significant (Table 1). This change parallels this species being morphologically and ecologically derived relative to the rest of this clade. In contrast to the other species of its clade, which are burrowers, this species is a crevice dweller, a niche convergently evolved with species such as *V. glauerti* which also is not resistant.

#### 2.3.7. Patterns of Venom Resistance in Varanid Orthosteric Sites and Reciprocal Patterns of Selective Actions by *Acanthophis* Venoms on Varanid Orthosteric Sites

**Figure 8:**
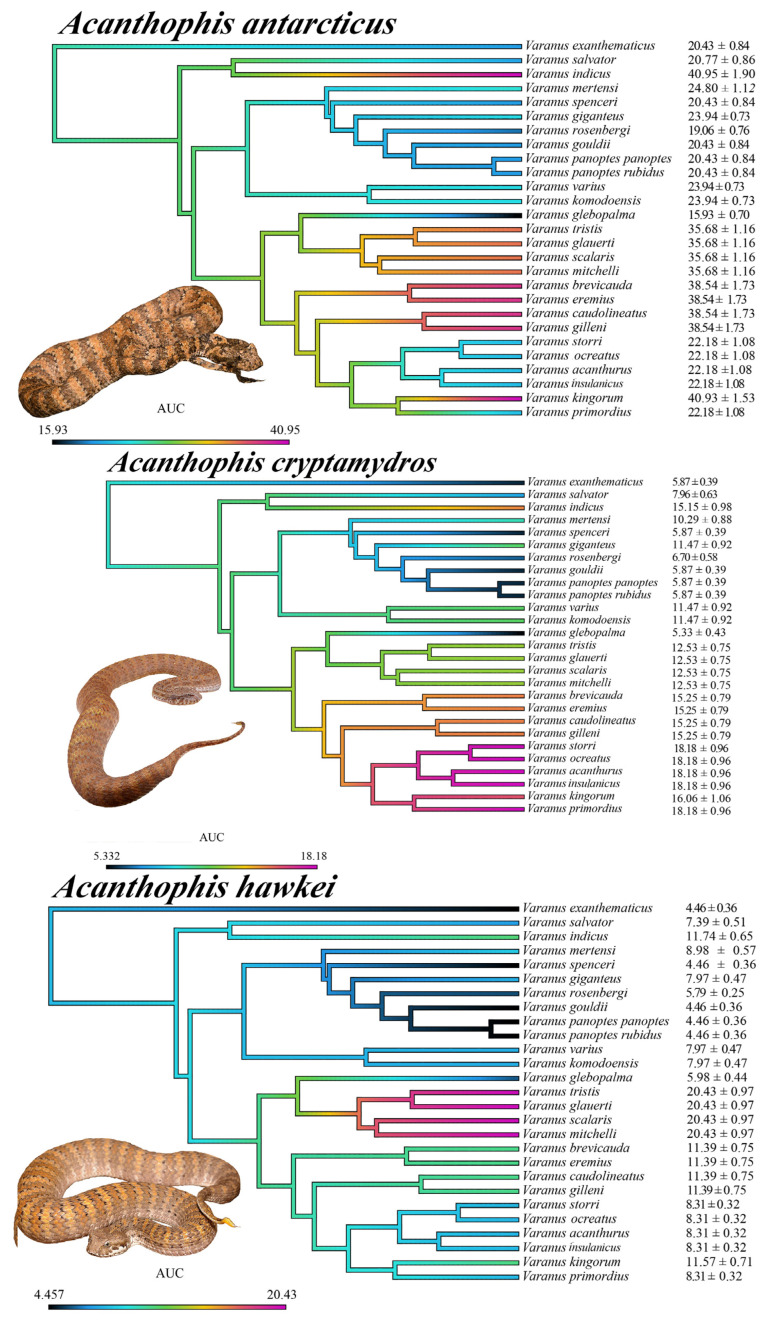
Ancestral state reconstructions of binding affinities to varanid orthosteric sites. All values are mean area under the curve (AUC) values ± standard deviation (n = 3). The color gradient ranges from violet to black, with violet representing strong α-neurotoxin binding affinities (larger AUC values) and black representing weak α-neurotoxin binding affinities. The phylogeny was produced using timetree.org (accessed on 3 September 2023) and updated in Mesquite software (v 3.81). Photo credits: CJHay (*A. cryptamydros*, *A. hawkei*); CNZdenek (*A. antarcticus*).

**Figure 9:**
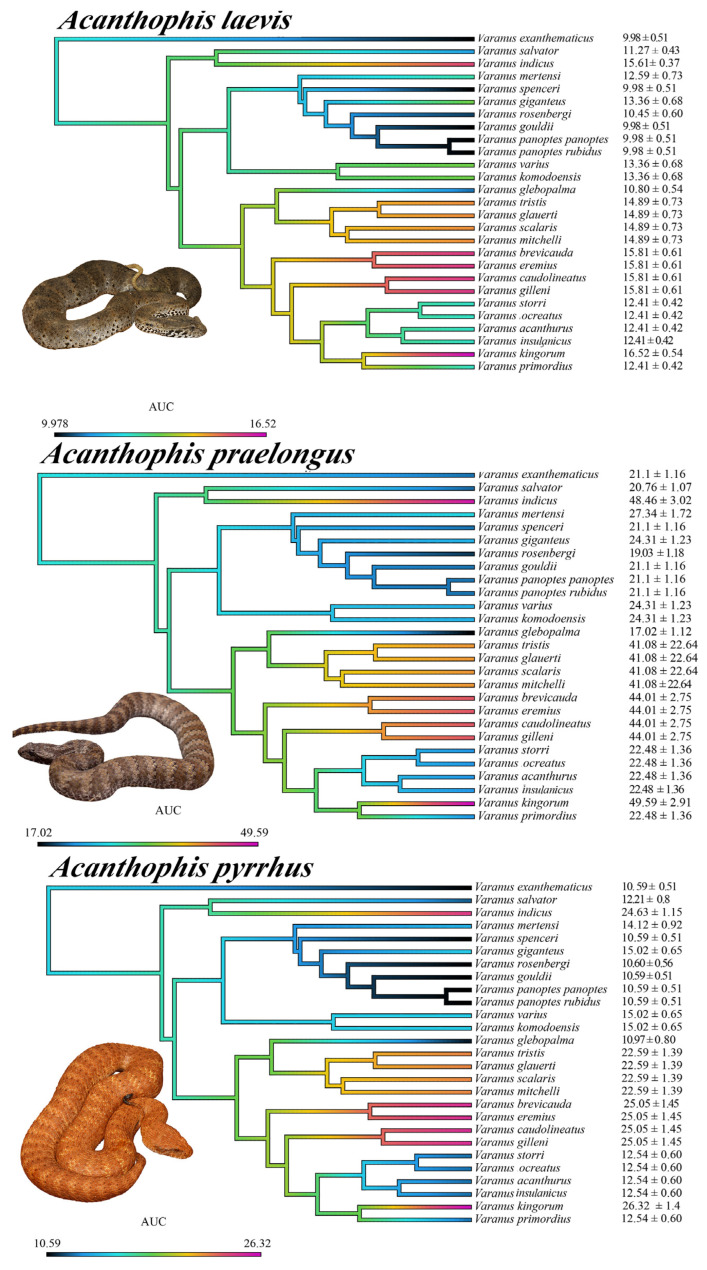
Ancestral state reconstructions of binding affinities to varanid orthosteric sites. All values are mean area under the curve (AUC) values ± standard deviation (n = 3). The color gradient ranges from violet to black, with violet representing strong α-neurotoxin binding affinities (larger AUC values) and black representing weak α-neurotoxin binding affinities. The phylogeny was produced using timetree.org and updated in Mesquite software. Photo credits: CJHay (*A. pyrrhus*); CNZdenek (*A. praelongus*, *A. rugosus*); Mark O’Shea (*A. laevis*).

**Figure 10:**
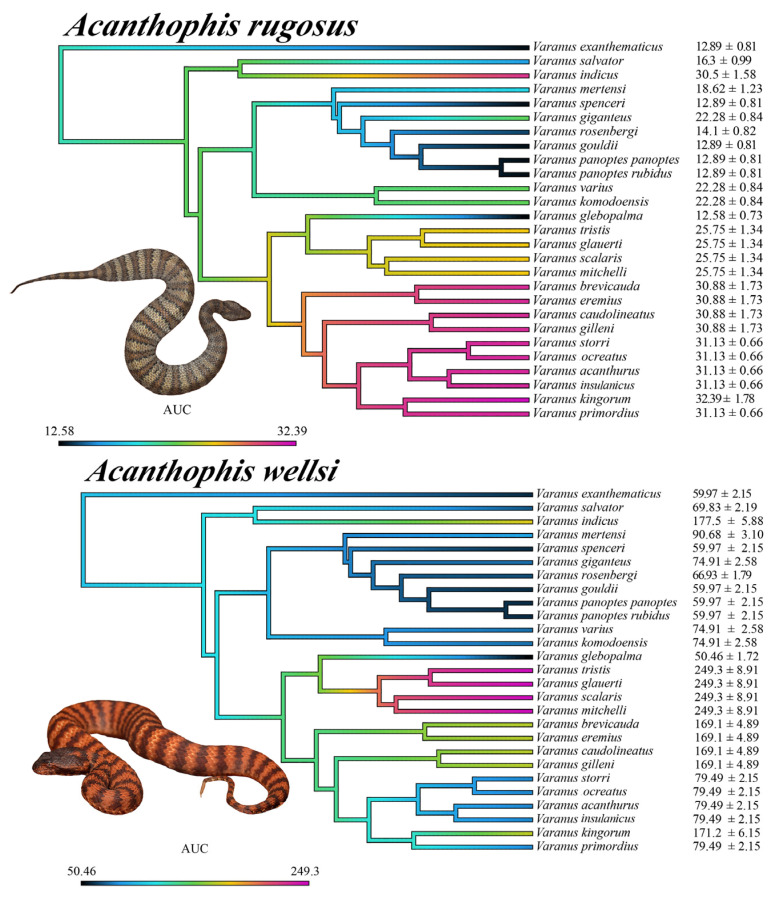
Ancestral state reconstructions of binding affinities to varanid orthosteric sites. All values are mean area under the curve (AUC) values ± standard deviation (n = 3). The color gradient ranges from violet to black, with violet representing strong α-neurotoxin binding affinities (larger AUC values) and black representing weak α-neurotoxin binding affinities. The phylogeny was produced using timetree.org and updated in Mesquite software. Photo credits: CJHay (*A. wellsi*); CNZdenek (*A. rugosus*).

**Figure 11:**
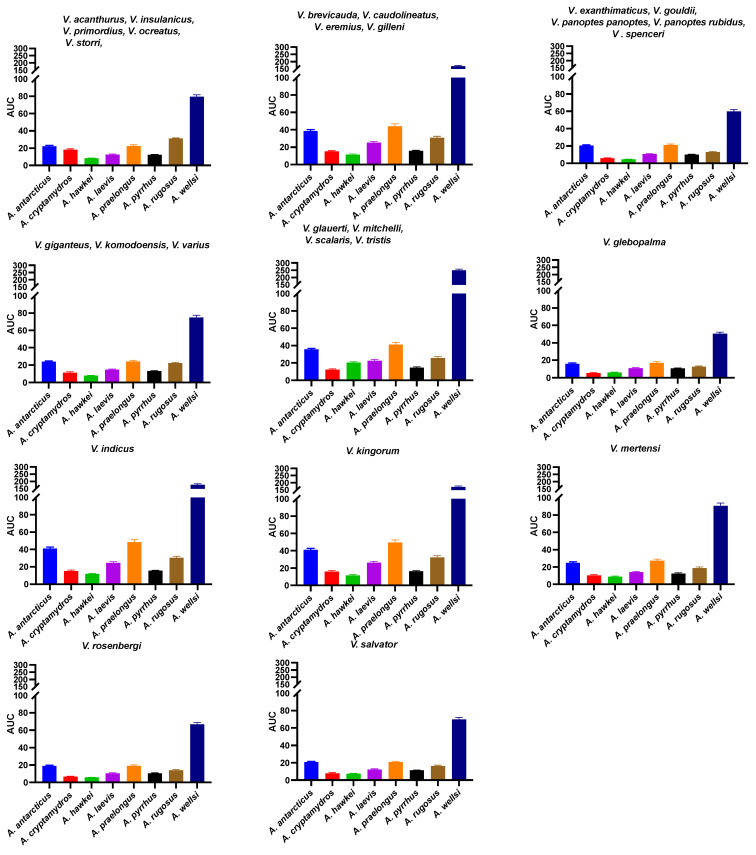
Bar graphs of the area under curve indicates comparison of venom binding affinities of the eight *Acanthophis* species’ venoms against native varanid mimotopes.

The above results collectively suggest that venom resistance was present in the last common ancestor of all varanid lizards. Within the large varanid lizards, it was secondarily lost in *V. indicus*. Within the dwarf varanids venom resistance has been a very dynamic trait, with it being secondarily lost in the last common ancestor of the clade consisting of *V. glebopalma*/*V. primordius* (Figure 3), subsequently regained on two separate occasions (*V. glebopalma* and again in the last common ancestor of the clade consisting of *V. storri*/*V. primordius* (Figure 3), and then lost again in *V. kingorum*. Tests showed these secondary derivations to be significant across all venoms (Table 1). There was however the conspicuous exception of the *V. storri/V. primordius* clade for *A. rugosus* venom (and to a lesser extent *A. cryptamydros* venom), with these uniquely potent against all members of this clade (Figure 8, Figure 9, Figure 10 and Figure 11), not just *V. kingorum*, suggestive of reciprocal adaptation by the snakes against the resistance exhibited by the non-*kingorum* members of this dwarf varanid clade. 

As such, our evaluation of the α-neurotoxin binding affinities for each mimotope against the venoms from eight species of the *Acanthophis* genus (death adders), which are sympatric with the varanids, revealed a distinct pattern of binding efficacy (Figure 8, Figure 9, Figure 10 and Figure 11). The results demonstrated that all the *Acanthophis* venoms bound more effectively to the native mimotopes of dwarf varanids compared to those of larger varanids. This pattern suggests that larger varanid species have evolved greater resistance to α-neurotoxins as the basal trait, while smaller species exhibit a comparatively lower resistance level as secondary derivations.

Ancestral state reconstruction (Figure 8, Figure 9 and Figure 10) demonstrated that the binding patterns of most *Acanthophis* species were highly similar. As noted above, there was however a conspicuous variation for *A. rugosus* which was notably more potent against the sympatric clade consisting of *V. storri*/*V. primordius* (Figure 10 and Figure 11, Table 1), which is suggestive of a reciprocal evolution of venom selectivity overcoming the resistance these species show against other *Acanthophis* venoms. This pattern was also evident, but to a lower degree of variation for *A. cryptamydros* (Figure 8 and Figure 11). Another notable variation was *A. wellsi* exhibiting dramatic increases in both the speed of action and overall potency relative to all other *Acanthophis* venoms tested (Figure 10 and Figure 11). Death adders have venoms rich in neurotoxic 3FX peptides [44], this means rather than *A. wellsi* venom being faster acting and more potent due to an increased presence of 3FTx, that the increases in both traits are due to biochemical modifications of the 3FTx present. Future efforts should focus on comparing the structure-activity relationships of *A. wellsi* to other *Acanthophis* venoms to unravel the changes that confer these advantages. These results suggest that the selection pressure to develop faster acting and more potent 3FTxs in *A. wellsi*’s venom may be due to the extremely arid and harsh environment (the Pilbara region of Western Australia) for which this species is specialised to occur, where food is scarce and thus there is an extreme selection pressure for predatory success.

### 2.4. Selection Pressure and Adaptive Evolution

We employed codon-based models in PamlX (v.1.3.1.) [45] using the software CodeML (v1.3.1) to infer positive selection. These analyses allowed us to examine the likelihood of adaptive evolution at the nAChR orthosteric site. We tested multiple approaches on different partitions of the dataset. First, a one-ratio model was implemented in order to estimate a single selection coefficient over the entire varanid taxa set. Secondly, we used branch models to infer selection patterns on two subpartitions of the dataset to assess how size and ecology might have shaped evolutionary patterns of toxin resistance at molecular level (see Section 4 for methods). The two different sets of foreground branches consist of (I) varanids averaging less than 1 m in adult size, and (II) a set of larger monitor lizards. 

The results of the one-ratio model indicated that the orthosteric region in varanid lizards is evolving under positive selection, yielding a selection coefficient ω of 1.0231 (Table 2). This result is already quite significant, as the sequence of the bioactive site of the nAChR would be expected to be highly conserved due its crucial role as a neural receptor. Furthermore, branch models revealed that while dwarf varanid lizards are evolving under purifying selection (ω = 0.8237), large ones are evolving under a large positive selection framework (ω = 1.4107; Table 2). These findings suggest that larger species feeding on α-neurotoxic snakes exhibit a strong trend toward sequence diversification in response to the exposure to venomous snake prey. On the other hand, smaller species were shown to be evolving under more conservative conditions, possibly due to the reduced interactivity with venomous snakes. Our findings, therefore, suggest that the driver of diversification within the binding site of the α-1 subunit should be considered as further evidence of how predator–prey arms races can shape evolutionary pathways. In addition to testing branch/clade evolution, we tested the individual codon-encoded sites for positive selection in both the above-mentioned models. The probability threshold used to consider a site to be under positive selection is the 95% criterion described in [45]. The Bayes Empirical Bayes outputs indicated positions 187 (96.1%), 189 (95.2%), 194 (95.8%), 195 (97.6%) (See Figure 3 for alignments at these positions) to be under positive selection across varanids. Mutations to these sites have all been identified to be involved in various means of resistance [4,5,16,22,26,28]. 

To further highlight how diversification within the α-1 subunit is restricted to the toxin-binding site, we performed the same analyses on the immediately flanking regions of the α-1 subunit, corresponding to positions 182 to 186, and 201 to 205, respectively. Results of the one-ratio model on the flanks indicated that these regions are evolving under strong purifying selection (ω = 0.1791; Table 2), with no sites found to be under positive selection either. This appears to be a further indication of positive selection being restricted to the orthosteric region of the receptor exclusively, the only region under pressure to evolve fitness-increasing mechanisms to cope with alpha-neurotoxin binding threat. Despite this, when the dataset was again subpartitioned into small and large species, we got interesting results. Consistent with the analyses on the orthosteric region, the flanking regions of both dwarf and large varanid sequences were found to be under extreme purifying selection (ω for small varanids = 0.0001 and ω for large varanids = 0.3245, respectively; Table 2). 

Subsequently, we tracked the origin of each modification thought to be associated with reduced toxin binding to the receptor using Mesquite (v.3.81) [46]. The character analyses were operated under the parsimony criterion (ACCTRAN method, which traces the origins as close as possible to the root). Analyses revealed a myriad of modifications occurring at orthosteric site 187, 189, 191, 194, 195, 196:

- Orthosteric site position 187:
  ○ Two origins of deviation from the ancestral tryptophan were found:
    ▪ *V. rosenbergi*, where it is replaced by a serine.
    ▪ *V. indicus* has glutamic acid at this position and *V. salvator* has glutamine. Considering the phylogenetic relatedness of these species (Figure 3), the most parsimonious explanation is a mutation to either glutamic acid or glutamine occurred in their last common ancestor, and that a subsequent mutation occurred to convert the derived amino acid into the other form. Further sequencing will be necessary, however, to reconstruct the molecular evolutionary history. Regardless, the change in venom affinity was shown to be statistically significant (Table 1).
- Orthosteric site position 189:
  ○ Threonine was found to be the ancestral condition.
  ○ A derivation to positively charged amino acid occurred at the base of the dwarf varanids (Figure 3). Subsequently this position was mutated into arginine in the last common ancestor of the *V. tristis*/*V. mitchelli* clade (Figure 3).
- Orthosteric site position 191:
  ○ The ancestral state was found to be represented by the achiral proteinogenic amino glycine.
  ○ Consequently, the negatively charged aspartic acid derived state, convergently evolved on three occasions (Figure 3):
    ▪ Last common ancestor of the *V. brevicauda/V. primordius* clade.
    ▪ *V. giganteus*.
  ○ Last common ancestor of the *V. komodoensis*/*V. varius* clade.
- Orthosteric site position 194:
  ○ A derivation from the ancestral proline occurred in the last common ancestor of the *V. glebopalma*/*V. primordius* clade.
  ○ Subsequently a secondary reversal occurred in *V. glebopalma* resulting in the reversal to the resistant state. The change in venom affinity was shown to be statistically significant (Table 1).
- Orthosteric site position 195:
  ○ Derivation from the ancestral asparagine (R) to aspartic acid (D) convergently occurred on three occasions (Figure 3):
    ▪ V. mertensi
  ○ Last common ancestor of the *V. glebopalma*/*V. primordius* clade.
    ▪ Subsequent reversal in *V. glebopalma*, resulting in the reversal to the resistant state. The change in venom affinity was shown to be statistically significant (Table 1).
    ▪ Subsequently in the *V. brevicauda*/*V. gilleni* clade there was a further derivation to glutamic acid (E).
- Orthosteric site position 196
  ○ A derivation from the ancestral threonine (T) to isoleucine (I) occurred *V. kingorum* and is associated with the loss of venom resistance in this species. The change in venom affinity was shown to be statistically significant (Table 1).

## 3. Discussion

The orthosteric sequences in larger varanids, paired with ligand-receptor interaction insights, point to a trend: the presence of neutral amino acids at key bioactive sites, especially at position 195, could be moderating α-neurotoxin resistance in these larger species. Supporting this, recent studies have shown that the negatively charged amino acids, aspartic acid (D) at position 191 and glutamic acid (E) at position 195, increase the affinity of positively charged 3-finger toxins (3FTxs) for the α1 nAChR orthosteric site through electrostatic attraction [2,22,23,24]. This contrasts with the substitution of these amino acids with neutrally or positively charged counterparts, which seems to offer resistance to neurotoxic snake venom.

Notably, larger Australian varanids, known to feed on venomous snakes like death adders (*Acanthophis* spp.), which have venom rich in postsynaptically acting α-neurotoxins, may have evolved orthosteric site residues that provide added protection against envenomations by neurotoxic snake prey species. Research indicates a synergistic role for aspartic acid (D) and glutamic acid (E) at these positions, possibly intensifying neurotoxin binding [2]. The significant binding seen in dwarf varanid species might be partially attributed to the presence of glutamic acid (E) at position 195, suggesting its importance in conferring neurotoxin resistance.

The Anguimorph genus *Varanus* evolved between 28 and 35 million years ago, with subsequent rapid radiation and diversification. This diversification led to their widespread distribution across Asia and Australia, with large-bodied varanids in Africa and Asia potentially evolving in response to competition with large mammals and coexistence with venomous snakes like cobras. This contrasts with Australian varanids that diversified in the absence of placental carnivores, filling ecological niches and adopting various predatory roles. Australian varanids are notably diverse, with about 30 endemic species adapted to a range of environments, characterized by distinct morphological and ecological features. These varanids demonstrate remarkable evolutionary adaptability, taking on roles from arboreal specialists to formidable predators, and are generally terrestrial and carnivorous. Australian varanids, in particular, have become apex predators, potentially driving the evolution of traits to resist α-neurotoxins due to their position in the predator–prey arms race.

Our findings indicate that larger varanids from Australia, Africa, and Asia have as predators evolved resistance to elapid venom α-neurotoxins. Conversely, Australian dwarf varanids as potential prey have shown variable resistance, suggesting they have been subject to different evolutionary pressures. While larger varanids demonstrated a consistent pattern of resistance, this was not a uniform trait among their Australian dwarf counterparts, pointing to diverse selective pressures shaping their resistance to neurotoxins. This highlights the complex interplay between evolutionary forces and environmental factors in shaping the survival strategies of these adaptable reptiles. 

The study uncovered a distinct survival advantage in larger varanids, revealing their enhanced resistance to α-neurotoxins conferred by the presence of specialized motifs within their nAChR orthosteric sites. This resistance is reflected in their ecological behaviors and interactions with venomous snakes, suggesting an evolutionary adaptation for survival against neurotoxic threats from venomous prey. An interesting trend was that the resistance was strongest in the mid-sized range of the Australian large varanids (*V. gouldii*, *V. panoptes*, and *V. spenceri*)*,* and, with a decrease in the largest species, with a reduction in resistance occurring convergently in the two clades which independently evolved extreme morphotypes: *V. giganteus* and again the last common ancestor of *V. komodoensis*/*V. varius* (Figure 3 and Figure 5). The change in venom affinity was shown to be statistically significant (Table 1). This is suggestive of mechanical means of defense against dangerous prey (thicker scales with larger osteoderms) and more effective predation (larger body size and teeth relative to the size of the prey). Thus, the selection pressure for venom resistance would be reduced in the largest species, which is also congruent with the ‘use it, or lose it’ pattern noted previously for resistant species, suggesting a fitness disadvantage imposed by resistance motifs resulting in less efficient binding by the endogenous neurotransmitter acetylcholine [25]. In the case of *V. indicus*, the unique ecological niche occupied by this species is one that would have few α-neurotoxic prey items [47,48]. Intriguingly, in contrast to the reduction of venom resistance in the largest Australian species, the equally massive Asian water monitor, *V. salvator* did not demonstrate a corresponding reduction in venom resistance paralleling the large morphotype. However, this species occurs sympatrically with abundant large α-neurotoxic elapid species in the *Naja* genus. This in contrast to the largest species of elapid snakes in Australia being presynaptically neurotoxic (e.g., *Oxyuranus*) or coagulotoxic (e.g., *Oxyuranus*, *Pseudechis*, *Pseudonja)* instead of α-neurotoxic, while the most potently Australian neurotoxic species (e.g., *Acanthophis*) are proportionally much smaller. Further natural history research is needed to confirm the level of pressure from neurotoxic elapid prey, and the efficiency of larger osteoderms as a mechanical form of protection. 

The relative level of venom resistance in Australian dwarf monitors was even more dynamic in their relative resistance to α-neurotoxic venoms, with a strong correlation to habitat specialization. For example, there was a strong variation in orthosteric site biochemical composition and relative levels of venom resistance between two terrestrial burrowing clades. High levels of resistance were noted for the hard-pack soil-type specialist clade consisting of *V. acanthurus*, *V. insulanicus*, *V. primordius*, *V. ocreatus*, and *V. storri*. In contrast, low levels of resistance were noted for the fine-sand soil-type specialist clade consisting of *V. brevicauda* and *V. eremius*. Paralleling the habitat specialization and relative levels of neurotoxicity sensitivity, is the relative presence of α-neurotoxic elapid snakes, with larger and more abundant species present in the hard-pack soils than in the fine-sand dunes. This is suggestive of differential selection pressures being exerted upon the two clades of terrestrial burrowing dwarf varanids. In contrast to the variations noted between the two clades of terrestrial specialists, resistance was not noted for any of the two convergent arboreal specialist clades; *V. caudolineatus*/*V. gilleni* clade; and for the *V. glauerti*, *V. glebopalma*, *V. mitchelli*, *V. scalaris*, *V. tristis* clade. Arboreality appears to be linked to low levels of venom resistance, suggestive of reduced predatory pressure by sympatric α-neurotoxic venomous snakes. Consistent with this, is the increased levels of resistance noted for *V. glebopalma*, which parallels the orthosteric site reverting to the basal traits of 194P and 195N (Figure 3 and Figure 5), and this species occupying a terrestrial niche in contrast to the arboreality of other members of its clade (*V. glauerti*, *V. mitchelli*, *V. scalaris*, *V. tristis*). The change in venom affinity was shown to be statistically significant (Table 1). As with the large varanids, the differential levels of venom resistance are linked to relative levels of interactions with α-neurotoxic venomous snakes, although in this case are suggestive of as prey of venomous snakes rather than as predators. The secondary losses, and gains, are consistent with resistance motifs coming at the cost of reduced fitness, underscoring the ‘use it or lose it’ selection pressure. This is exemplified by *V. kingorum* evolving as a crevice specialist from within the burrowing *V. acanthurus*/*V. storri* clade, with the loss of the resistance which had been regained in the *V. acanthurus*/*V. storri* clade after the loss at the base of the dwarf clade. This loss of resistance parallels the loss of resistance in other crevice dwellers such as *V. glauerti*. The change in venom affinity was shown to be statistically significant (Table 1).

These evolutionary trends illustrate the complex interplay between predator–prey dynamics and the molecular evolution of toxin resistance mechanisms within these lizard species. Consistent with this was the increased potency for the venoms of *A. cryptamydros* and *A. rugosus* for sympatric varanid species, suggestive of these varanids could be experiencing predatory pressures from these specific elapids. This scenario is similar to that seen in other neurotoxic snakes like the King Cobra (*Ophiophagus hannah*), which possess venoms highly potent to their snake prey [2]. Such a relationship suggests a nuanced interplay between predator and prey, with varanids’ resistance mechanisms and snakes’ venom potency being shaped by each other’s presence and ecological strategies. This interplay may provide significant insights into the diet-related adaptive evolution of snake venom systems and the selective pressures exerted by prey pathophysiological targets. However, to fully elucidate these complex ecological and evolutionary dynamics, further dietary and behavioral studies on these species are warranted. 

In conclusion, our data underscore the evolutionary pressures leading to toxin resistance in larger varanids, likely driven by predation on neurotoxic elapids. The limited interaction between smaller Australian varanids and neurotoxic elapids, coupled with their dietary habits that involve preying on juvenile snakes, results in a lesser evolutionary force acting upon their receptor sites. This is reflected in the reduced number of resistance motifs and increased vulnerability to α-neurotoxins in comparison to their larger counterparts. This investigation contributes valuable insights into the adaptive evolution of varanids, highlighting the influence of predator–prey dynamics on their physiological evolution. Despite these advancements, our understanding is curtailed by the current insufficiency of detailed natural history and dietary data for these lizards. Addressing this knowledge gap through focused future research remains imperative for a comprehensive appreciation of these intricate evolutionary processes. In addition to further work investigating varanid lizard resistance to other venomous animals (whether as predators or prey), future work should also investigate the resistance of Anguimorph lizards to their own venoms, including presynaptic ion-channel toxins [49], and toxins that act upon the blood clotting cascade [50,51,52].

## 4. Materials and Methods

### 4.1. DNA Extraction from Varanid Tissue Samples

For DNA extraction, tissue samples of Australian Monitor lizards from the family Varanidae were obtained from collaborators of the Australian Museum (Sydney Australia) and the Museums Victoria (Melbourne, Australia) via tissue specimen loans. Details are shown in Supplementary Table S1. These samples represent the full ecological, morphological, and taxonomical diversity of Australian varanid lizards, from the smallest (*V. brevicauda*) to the largest (*V. giganteus*) and from burrowing (*V. brevicauda* and *V. eremius*) to arboreal (*V. scalaris*) species. DNA was isolated from the varanid tissue samples with DNeasy Blood & Tissue kit (QIAGEN, Carlsbad, CA, USA) using an optimized spin column-based protocol according to the manufacturer’s instructions. Since the varanid tissue samples were preserved in either 70% ethanol or DMSO/alcohol/NaCl solution mixture, the tissues were rinsed with 10% phosphate-buffered saline (PBS) prior to the extraction. Around 25 mg of finely segmented tissue samples from each of the varanid tissue were taken for extraction and mixed with lysis buffer and Proteinase K solution. The mixtures were incubated for around 3 h for complete lysis with gentle shaking at 56 °C in a digital heat block. After the incubation, tissue lysates were subjected to DNA precipitation in absolute ethanol following further lysis and then subjected to a series of centrifugation steps with washing buffers. After carrying out the final elusion, the concentration and the purity of the eluted DNA were determined using the Nanodrop 2000 UV–VIS Spectrophotometer (Thermo Fisher Scientific, Waltham, MA, USA). All centrifugation steps were carried out at room temperature. Isolated varanid genomic DNA was stored at −20 °C.

### 4.2. Amplification of Orthosteric Site Sequence of α-1 Subunit of nAChR 

The primer-directed PCR amplification was used to amplify a short (~200 base pairs) stretch containing the ligand binding pocket of the α-1 subunit of the muscular nAChR gene (chrna1). For this, amplification primers containing locus-specific components were used. Primers specific for the orthosteric site of the nAChR were designed on the alignment of the reference sequence of the species of *Varanus komodoensis* (Komodo dragon) obtained from the National Centre of Biotechnology Information (NCBI) database (*Varanus komodoensis* strain SLA01 scaffold18_1, whole genome shotgun sequence) Accession: SJPD01000023.1 39. PCR reaction conditions were optimized in several trials with the manufacturer’s instructions (Taq PCR master mix kit QIAGEN). The reaction volume of each of the PCR reaction mixtures was set up to 50 μL. The pair of locus-specific primers used in the present study were:

Var_Loc_F1 TAAGTAACTACATGGAGAGTGG,
Var_Loc_R1 TGTGGGCAGATAAAAGACTAAACC
whilst the primer annealing temperature was set to 55 °C.

### 4.3. Sequencing of nAChR

The amplified PCR products were subjected to dual-direction sequencing using the automated dideoxy sequencing method (Australian Genome Research Facility, Brisbane, Australia) and the resulting sequence reads were then assembled, aligned and manually curated using software Aliview v.1.1 (alignment viewer and editor) and Expasy Translate Tool (https://web.expasy.org/translate/, accessed on 5 November 2023)) to identify the presence or absence of resistance elements in the ligand-binding domain of the α1subunit of nAChR of each of the varanid species tested. Genbank accession codes for sequences obtained in this study are: *Varanus acanthurus* PP333912, *V. brevicauda* PP333906, *V. caudolineatus* PP333908, *V. eremius* PP333911, *V. exanthematicus* PP333904, *V. gilleni* PP333907, *V. glauerti* PP333917, *V. glebopalma* PP333905, *V. gouldii* PP333925, *V. indicus* PP333920, *V. insulanicus* PP333913, *V. kingorum* PP333914, *V. mitchelli* PP333916, *V. ocreatus* PP333909, *V. panoptes* PP333924, *V. panoptes rubidus* PP333923, *V. primordius* PP333915, *V. rosenbergi* PP333921, *V. scalaris* PP333919, *V. spenceri* PP333926, *V. storri* PP333910, *V. tristis* PP333918, and *V. varius* PP333922. Genbank accession codes for previously obtained sequences are: *V. exanthematicus* SRA:SRR6144715.1034956.2, *V. giganteus* MT249131.1, *V. mertensi* MT249118.1, *V. komodoensis* MT249129.1, and *V. salvator* JAIXND010000755.1 regions 56973721 to 56973762.

### 4.4. Venom Stock Collection and Preparation

Pooled venom samples from eight species of death adders (genus *Acanthophis*) spanning across multiple geographic locales within Australia were sourced from the long-term cryogenic collection of the Adaptive Biotoxicology Lab, University of Queensland, St Lucia, Australia. All the venom study protocols of this work were performed with University of Queensland Biosafety Approval #IBC134BSBS2015 and University of Queensland Animal Ethics Approval 2021/AE000075. The lyophilized crude venom samples were reconstituted with double-deionized water (ddH_2_O) prior to use. The centrifugation was performed at 14,000 RCF for 10 min with a temperature of 4 °C. Subsequently, the pellet, (if any), was discarded, and the supernatant was used to make a working venom stock of 1 mg/mL in 50% of glycerol in order to preserve the enzymatic action while avoiding freezing upon storage at −20 °C. The concentrations of the prepared venom stocks were checked at 280 nm with a NanoDrop 2000 UV–VIS Spectrophotometer (Thermo Fisher Scientific, Waltham, MA, USA). 

### 4.5. Mimotope Design and Preparation 

A series of 14-amino-acid-long short peptide mimotopes, which represent the region of 187–200 of α-1 subunit of muscle-type nAChR of varanids were designed following the previously validated protocols [2,23,24,42,43,53,54,55,56], including the cysteine-doublet substituted during peptide synthesis by a serine doublet to prevent uncontrolled postsynthetic thiol oxidation [41]. The amino acid sequences of orthosteric sites except for the two sequences of *V. komodoensis* and *V. salvator* were obtained from the sequencing data of the present research work. The α1 orthosteric site amino acid sequence *V. komodoensis* was from NCBI accession SJPD01000023.1 39) and the *V. salvator* was the NCBI genome section JAIXND010000755 56973721 to 56973762. Another series of varanid mutant mimotopes were designed by substituting the identified key residues with corresponding orthosteric positions. The C-C bond which is localized at positions 192 and 193 of each of the mimotopes was replaced with S-S bond in order to avoid the uncontrollable postsynthetic thiol oxidation. Since the highly conserved cysteine (C) residues do not directly participate in receptor–ligand interactions, the elimination of the two tandem cysteine residues from the ligand binding region does not produce a considerable effect on the ligand binding. However, it has been identified that the presence of cysteines plays an important role in the structural formation of the ligand-binding site of the overall receptor. Therefore, the ligand-binding kinetics of the whole receptor and the synthetic peptide mimotopes are not exactly comparable or should be interpreted carefully. Working mimotope stock solutions of 50 µg/mL were made by dissolving each of the supplied mimotopes of dried pellet in 100% dimethyl sulfoxide (DMSO) following 1:10 dilution with double-deionized water. All prepared mimotope stock solutions were stored at −20 °C for future use. 

### 4.6. Biolayer Interferometry Assay (BLI)

The receptor-neurotoxin binding affinities were measured with a label-free, microfluidics-free novel biolayer interferometry assay, which was performed on an Octet HTX system (ForteBio, Fremont, CA, USA). All the experimental procedures of the octet potency assay along with the data acquisition and data analysis were performed according to the previously validated protocol [43]. The increased accumulation of ligand molecules at the interacting surface increases the optical thickness of the biosensor which results in changes in the light reflection pattern from the biosensor surface. This produces a quantifiable spectral shift, which will consequently result in binding kinetic information that can be quantified in real-time. The final concentration of the analyte sample was set at 50 µg/mL per well via diluting the venom stock solution in a 1:20 dilution ratio with Dulbecco’s phosphate-buffered saline (DPBS) with 0.1% BSA and 0.05% Tween-20. The stock solutions of each of the mimotopes were diluted up to the final concentration of 1 µg/mL per well with a ratio of 1:50 in Dulbecco’s phosphate-buffered saline (DPBS). Before use in the biolayer interferometry assay, streptavidin biosensors were hydrated in Dulbecco’s phosphate-buffered saline (DPBS) at room temperature for 30 min with gentle rotation at 2.0 revolutions per minute (RPM). The standard acidic glycine buffer solution (10 mM glycine (pH 1.5–1.7) in ddH_2_O) was used as the dissociation buffer in order to detach the bound analytes from the interacting surface of the streptavidin biosensor. Raw data are in the Supplementary Materials Datasheets for Figure 5, Figure 6, Figure 7, Figure 8, Figure 9, Figure 10 and Figure 11. 

### 4.7. Data Acquisition, Processing, and Statistical Analysis 

Snout-vent length (SVL) measurements for all varanid species tested were retrieved from relevant literature and imported into GraphPad PRISM 9.2 (GraphPad Software Inc., La Jolla, CA, USA) and divided into two categories based on size, i.e., large (SVL > 500 mm) and dwarf (SVL < 500 mm) species [29,57]. The data were visualized in a box-and-whisker plot and an unpaired Welch’s *t*-test was performed to check for significance of the difference in mean SVL between the two groups. Normality of distribution and homogeneity of variance were assessed visually with a QQ-plot and a homoscedasticity plot, respectively.

All the data acquired from biolayer interferometry on an Octet HTX (ForteBio, Fremont, CA, USA) were processed in exact accordance with the previously validated protocol of the assay [43]. The raw data of the association step (performed in triplicate) were extracted from the Octet HTX system and were processed and saved as an Excel.csv file. The processed data were imported into Prism 9.2 software (GraphPad Software Inc., La Jolla, CA, USA), and calculations of the area under the curve (AUC) were calculated, while graphs were constructed. The ordinary one-way ANOVA with Tukey’s multiple comparisons test was performed, and all assumptions (normality of residuals and homogeneity of variance) were checked with QQ plots and Brown–Forsythe tests. 

### 4.8. Evolutionary Analysis

To analyze the evolutionary trends at the orthosteric region of the α1 subunit of the nAChR (positions 187 to 200), a dataset of 27 varanid sequences was analyzed in PAMLX (v.1.3.1) [45], using the program CodeML (v1.3.1). CodeML uses maximum likelihood to perform codon-based analyses on a multisequence alignment and the corresponding phylogenetic tree under a series of different frameworks. To strengthen our claims of positive selection only occurring at the toxin-binding region of the receptor, we compared this region to the immediately flanking sections (sites 182 to 186. and 201 to 205, respectively). Rates of evolution are measured as the ratio of non-synonymous (dN) to synonymous (dS) nucleotide substitutions in the sequence (dN/dS, commonly expressed as the selection coefficient “ω”). An ω < 1 indicates purifying selection, which is found at highly conserved sequences, where little-to-no deviation from the ancestral state occurs. ω > 1 represents positive selection. Finally, ω = 1 is a sign of neutral evolution, resulting from an equal amount of non-synonymous and synonymous changes at DNA level. The analyses in codeML were performed under two different frameworks: (A) a “one-ratio model”, in which a single selection coefficient ω is calculated across the entire dataset; (B) a “branch-model”, used to infer how different branch sets, partitioned on the tree file as described in [45], are evolving within the tree. Branch models require the specification of one or more foreground branches to be compared against the rest of the tree (background branches). The foreground branches consisted of (I) a clade of small-sized varanids averaging <500 mm in SVL at the adult stage, and (II) a clade of larger (>500 mm SVL) varanids. 

Additionally, site specification was implemented on both models (one-ratio and branch models) to estimate which sites are evolving under positive selection. Estimates of positive selection at site level were obtained through the Bayes Empirical Bayes (BEB) algorithm implemented within CodeML. Sites were identified under positive selection if above a 95% probability threshold [45]. The phylogenetic tree of varanids used for this analysis was downloaded from timetree.org [58], a portal providing time-calibrated phylogenies of given sets of taxa inferred from multiple studies. As some of the species analyzed in this study are absent from Timetree, these were manually added to the tree, and subsequently visualized on the editing software FigTree (v.1.4.4.). The evolution of each single trait associated with toxin resistance was then traced in Mesquite (v.3.81.) [46]. Character reconstructions were used to determine which amino acid residues were representative of the basal state at important bioactive locations as well as to pinpoint the nodes at where the change emerged.

### 4.9. Phylogenetic Tree Heat Maps

Following similar methodology to a previous study, a phylogenetic tree of the varanid species included in the study were exported from timetree.org and imported into Mesquite, where additional manual editing was performed [59]. To produce a phylogeny, Timetree collates data from published studies, although the resulting phylogenies are sometimes incomplete. Since some of the varanid species were missing from the phylogeny and added manually in Mesquite based on phylogenies. The updated phylogeny was then imported into the statistical software R (version 3.6.1) using the APE package (https://cran.r-project.org/web/packages/ape/index.html, accessed on 1 August 2023) [60]. The contMAP function in the phytools package (https://cran.r-project.org/web/packages/phytools/index.html, accessed on 1 August 2023) [61] was used to map the trait (*Acanthophis* venom binding affinity) over the phylogenetic tree by estimating ancestral states using maximum likelihood. The methods for the binding affinity assays are found above in the Biolayer interferometry assay (BLI) section. In total eight *Acanthophis* venoms were tested for their binding affinity to the orthosteric site of α-1 subunit of the nAChR of each of the varanid species, thus eight trees were produced—one for each of the death adder species’ venoms. Trees were exported from R and edited using Adobe Acrobat Pro DC (v2023.008.20470) and Adobe Photoshop (v25.3.1). Editing mainly included adding species names and adding the mean ± standard deviation of AUC values to each branch. 

## Figures and Tables

**Table 1:** Tests for significance relative to other species within the clade from which they evolved for the secondary derivations of regaining resistance (*V. glebopalma*) or reduction due to change in ecological niche (*V. indicus* and *V. kingorum*) or the evolution of gigantism *(V. komodoensis*, *V. giganteus*, and *V. varius*).

	*V. kingorum* vs. Ancestral Clade ^1^ *p* =	*V. indicus* vs. *V. salvator* *p* =	*V. glebopalma* vs. Ancestral Clade ^2^ *p* =	*V. komodoensis/V. varius* and *V. giganteus* ^3^ vs. Ancestral Clade ^4^ *p* =
***A. antarcticus***	0.0001	0.0007	<0.0001	0.0058
***A. cryptomydros***	0.0641	0.0010	0.0005	0.0036
***A. hawkei***	0.0071	0.0010	0.0003	0.0007
***A. laevis***	0.0007	0.0002	0.0020	0.0031
***A. praelongus***	0.0009	0.0017	0.0012	0.0301
***A. rugosus***	0.3472	0.0005	0.0006	0.0260
***A. pyrrhus***	0.0010	0.0002	0.0008	0.0010
***A. wellsi***	0.0005	0.0003	0.0004	0.0017

**^1^** *V. kingorum* ancestral clade species = *V. acanthurus*, *V. insulanicus*, *V. primordius*, *V. ocreatus*, *V. storri*. **^2^** *V. glebopalma* ancestral clade species = *V. glauerti*, *V. mitchelli*, *V. scalaris*, *V. tristis*. **^3^** *V. komodoensis* and *V. varius* share the same orthosteric site trait in their last common ancestor, while this sequence was convergently evolved in *V. giganteus*. ^4^ *V. komodoensis*/*V. giganteus*/*V. varius* *ancestral clade species = *V. gouldii*, *V. panoptes panoptes*, *V. panoptes rubidus*, *V. spenceri*.

**Table 2:** Patterns of natural selection at the orthosteric (toxin-binding) site of the α 1 subunit (positions 187–200) and the immediately flanking regions (positions 182–186 and 201–205). Listed ω values represent selection coefficients (ω). Values <1 indicate negative (purifying) selection, values close to 1 reflect neutral evolution, and values >1 indicate positive (diversifying) selection.

ω Values			
Selection Model	Clade/Branch Set	Toxin-Binding (Orthosteric) Site	Flanking Regions
One-ratio model	All varanids (27 spp.)	1.0231	0.1791
Branch model	“Small” varanids (SVL < 500 m)	0.8237	0.0001
	“Large” varanids (SVL > 500 m)	1.4107	0.3245

## Data Availability

All data is presented in figures and in Supplementary Materials and Datasheets.

## Supplementary Materials

The following supporting information can be downloaded at: https://www.mdpi.com/article/10.3390/ijms25052628/s1.
